# Cardiorespiratory effects of different intraabdominal pressures in sheep: An experimental study

**DOI:** 10.14814/phy2.15506

**Published:** 2022-11-10

**Authors:** Paulo R. L. do Nascimento, Liana V. de Gouvêa, Thiago R. S. Leite, André L. de Sousa Teixeira, Paulo C. A. R. da Silva, José A. D. F. Filho, Michel J. S. A. Helayel, Amary N. Júnior, Isabelle M. da Cunha, Júlia de Souza Mendonça, Pedro L. Silva, Daniel A. B. Lessa

**Affiliations:** ^1^ Department of Pathology and Veterinary Clinic, Faculty of Veterinary Medicine Fluminense Federal University Niterói RJ Brazil; ^2^ Laboratory of Pulmonary Investigation, Carlos Chagas Filho Biophysics Institute Federal University of Rio de Janeiro Rio de Janeiro RJ Brazil

**Keywords:** blood gas analysis, echocardiography, laparoscopy, pneumoperitoneum, respiratory mechanics

## Abstract

Increased intraabdominal pressure (IAP) during laparoscopy can reduce venous return, but changes in respiratory system mechanics and their effect in left cardiac function are not well documented. This study evaluated the effects of different IAPs on respiratory mechanics and cardiac function in 10 healthy nonpregnant adult Santa Ines ewes randomly submitted to a crossover study using different IAPs: 0 mm Hg (G1), 10 mm Hg (G2), 12 mmHg (G3), and 15 mmHg (G4). Animals were anesthetized and mechanically ventilated (*V*_T_ = 15 ml/kg; positive end‐expiratory pressure = 3 cmH_2_O; FiO_2_ = 1.0). Pneumoperitoneum was induced by Hasson's trocar cannula. Variables were measured at INITIAL (IAP, 0 mmHg) and FINAL time points for each IAP after 1 h. At FINAL, driving airway pressure (Δ*P*,_RS_), and percentage fraction of dead space (*V*d/*V*t) were higher in G3 and G4 than G1 (*p* = 0.002, difference in means [MD] 4.60, 95% CI: 7.91–1.28, and *p* < 0.001, MD 5.4, 95% CI: 8.7–2.0; *p* = 0.016, MD −9.5, 95% CI: −17.9 to −1.2; and *p* = 0.027, MD −8.7, 95% CI: −17.1 to −0.4). The ejection fraction and fractional shortening were lower in G3 (*p* = 0.039, MD −11.38, 95% CI: −0.07−−22.68; *p* = 0.015, MD −13.05, 95% CI: −1.74−−24.36) and G4 (*p* = 0.039, MD −9.94, 95% CI: −0.07 to −19.80; *p* = 0.015, MD −11.43, 95%CI: −1.57 to −21.30, respectively) than G2. In G3, the maximum pulmonary flow velocity correlated negatively with Δ*P*,_RS_ (*r* = −0.740; *p* = 0.018), and *V*d/*V*t correlated positively with Δ*P*,_RS_ (*r* = 0.738, *p* = 0.046). At IAP of 12 and 15 mm Hg impaired respiratory system mechanics, reduced left cardiac function and no change in maximum pulmonary artery flow velocity were detected. Therefore, respiratory mechanics should be monitored as an interplay to reduce left cardiac function.

## Graphical abstract

Impaired respiratory system mechanics, and reduced left cardiac function were observed at IAP of 12 and 15 mmHg, while no changes in maximum pulmonary artery flow velocity were detected. This demonstrates the importance of monitoring respiratory mechanics to preclude changes in left cardiac function. This experimental study may have an impact on those patients with baseline reduced cardiac function before laparoscopic procedures.

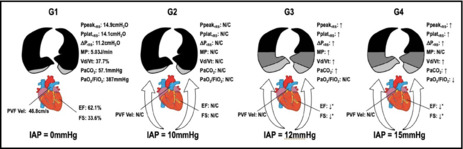

## 1 INTRODUCTION

Over 14 million laparoscopic procedures are performed globally every year.(iData Research Inc, 2020) During the laparoscopic procedure, intraabdominal pressure (IAP) must be increased to a certain level, for instance, greater than 12 mm Hg. Intraabdominal hypertension (IAH) is defined as the maintenance of IAP ≥12 mm Hg, even in the short term. IAH is associated with significant morbidity and mortality in surgical patients (Malbrain et al., 2005). The respiratory effects of increased IAP levels during laparoscopic procedures are well known and include hypercapnia, hypoxemia, and reduced respiratory system compliance (Atkinson et al., 2017). CO_2_ insufflation within the peritoneal cavity, as well as an increase in IAP, can promote changes in cardiorespiratory interaction and hydroelectrolytic and acid–base balance (Koivusalo & Lindgren, 2000). Reduced venous return is also expected after hours of exposure to increased IAP (Atkinson et al., 2017). However, the cardiopulmonary interaction as a consequence of increased IAP levels during laparoscopic procedures is not well documented. Both increased IAP levels and the amount of CO_2_ insufflation can constrict the pulmonary vasculature, which characterizes an acute dead space effect (Atkinson et al., 2017). Furthermore, it is widely known that impairment in respiratory system mechanics due to low chest wall compliance during increased IAP promotes changes in the venous pressure gradient from the inferior vena cava toward the right atrium. However, the contribution of respiratory system mechanics to left cardiac function during laparoscopic procedures with increased IAP values is not well understood. Therefore, we hypothesized that increased IAP levels may impair respiratory mechanics, which in turn can decrease venous drainage from the lungs toward the left side of the heart. This should be followed by a decrease in the left ventricular function, which may predispose patients to venous stasis and impair alveolar fluid homeostasis. Therefore, the aim of the present study was to evaluate respiratory mechanics and hemodynamic changes in sheep at four different IAP levels, using protocols similar to those used in surgical procedures in humans.

## 2 METHODS

### 2.1 Ethics

Ethical approval for this study (Ethical Committee N° 2115100418) was provided by the Ethical Committee on the Use of Animals of the Fluminense Federal University, Niterói, Brazil (Chairperson Prof. Mônica Diuana Calasans Maia) on August 9, 2018. In addition to the ethical guidelines established by the University's Committee, the experiment followed the ARRIVE guidelines described by Percie du Sert et al. (2020).

### 2.2 Animal preparation

Ten female nonpregnant healthy adult Santa Ines ewes (3.8 ± 1.2 years, 48.8 ± 5.6 kg) were clinically examined and kept in collective stalls, fed with chopped Napier grass (*Pennisetum purpureum*), commercial feed (300 g day^−1^ animal^−1^; 12% protein), water, and mineral salt ad libitum. Red blood cell, total protein, fibrinogen, and parasite control tests were performed. Seventy‐two hours before the experimental procedure, all animals received half of the food; 24 h before the procedure, they were fasted, and the water supply was suspended 6 h before, as recommended by Massone (2011).

### 2.3 Experimental design and protocol

All animals were subjected to the following pneumoperitoneum pressures: 0 mm Hg (G1), 10 mm Hg (G2), 12 mm Hg (G3), and 15 mm Hg (G4) in a crossover design (Table S1). The interval between each round of four animals was at least 15 days because this is the minimum time required for serum levels of malondialdehyde to return to the preoperative baseline values according to a pilot study conducted by our research group. The IAPs and times were chosen based on previous laparoscopic procedures in humans (Neudecker et al., 2002).

**FIGURE 1:**
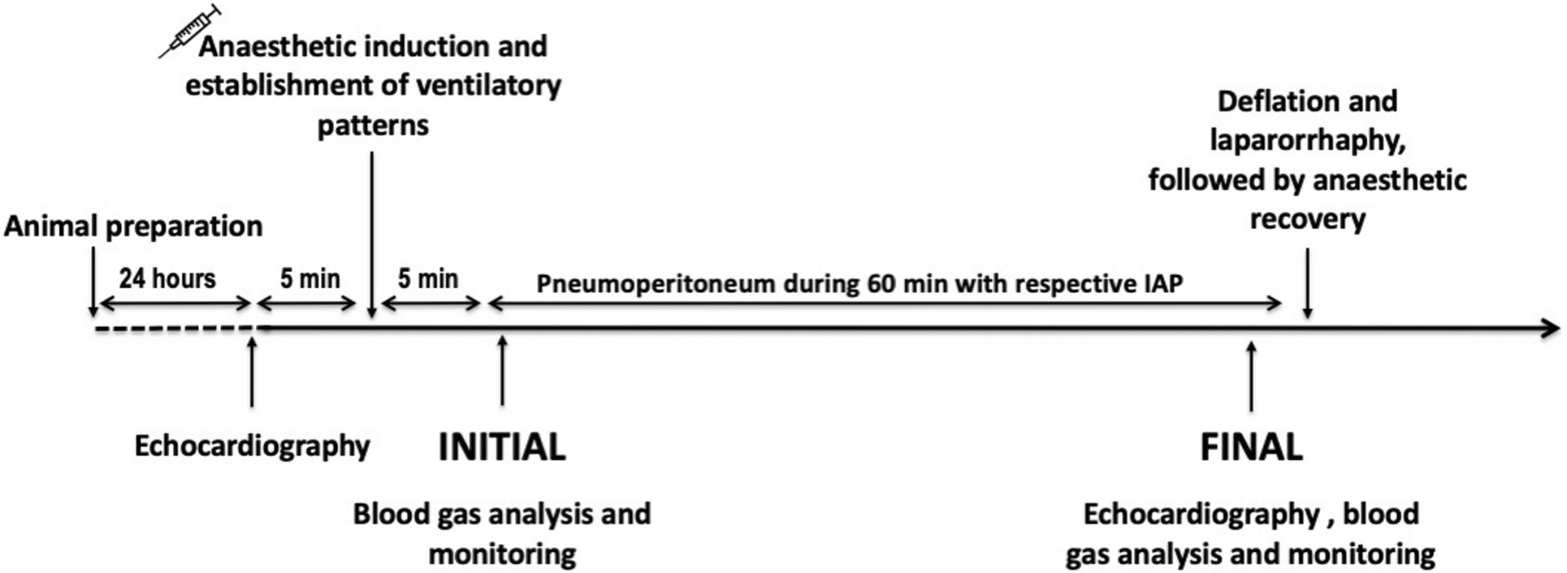
Experimental design.

A timeline of the procedure is shown in Figure 1. The animals were weighed before they were transferred to the operating room for echocardiographic examination at the start of the experiment (INITIAL; Figure 1). Anesthetic procedure was according to Rodrigues et al. (2017) and pre‐anesthetic medication was administered as follows: midazolam (0.3 mg/kg, Dormire, Cristália) and meperidine (3.0 mg/kg, União Química) in the same syringe intramuscularly, dipyrone (20 mg/kg, Ibasa) intravenously (IV), and citrate fentanyl (5 μg/kg, IV) through the jugular vein, using an 18G intravenous catheter. Continuous citrate fentanyl IV was initiated at a rate of 5 μg/kg/h using a syringe infusion pump (Digicare Biomedical Technology).

Pharmacologic induction consisted of 4 mg/kg propofol IV (Midfarma), and once a state of unconsciousness of the animal was clinically observed, the animal was placed in sternal recumbency and an endotracheal tube (Surgivet FN7; inner diameter, 7 mm; outer diameter, 10 mm; length, 55 cm) was inserted to maintain airway flow and connected to the circular valve anesthesia system with gas rebreathing and CO_2_ absorption by soda lime and a capnograph tube (Dameca Siesta Breasy) connected to the endotracheal tube. An inhaled anesthetic agent, isoflurane (Cristália), was used to maintain anesthesia, with a vaporized fraction between 1.5 and 2.5 vol.%. Tidal volume (*V*_T_) was adjusted to 15 ml/kg, positive end‐expiratory pressure (PEEP) was set at 3 cmH_2_O, mean respiratory rate (RR) adjusted to 8 breaths per minute (bpm), ranging between 6 and 10 bpm, as proposed by Massone.(Massone, 2011) Five minutes before peritoneal cavity insufflation with CO_2_, 0.3 mg/kg of atracurium besylate (Tracur, Cristália) was administered IV for neuromuscular blockade. After arterial blood gases and cardiovascular and ventilatory parameters were acquired at INITIAL, the animals were subjected to the following IAPs according to groups G1–G4.

Pneumoperitoneum was induced and maintained by positioning Hasson's trocar cannula attached to an insufflator (Electronic Endoflator 264305 20; Karl Storz). Thereafter, insufflation of the peritoneal cavity was gradually started toward the IAP in each group. After achieving the target IAP, all animals were rigorously subjected to 60 min of pneumoperitoneum. Subsequently, FINAL data collection was performed, and passive deflation occurred. In G1, the device was connected, and the pressure was maintained at 0 mm Hg.

Postoperative management consisted of analgesia with dipyrone IV every 24 h for 5 days and enrofloxacin 10% (Zelotril, Agener União) 2.5 mg/kg IV every 24 h for 7 days.

### 2.4 Cardiovascular and ventilatory monitoring

The cardiovascular and ventilatory parameters were assessed continuously throughout the experimental procedure (Multi‐Parameter Physiologic Monitor Lifewindow 6000; Digicare), and data were collected at the INITAL and FINAL time points. Specifically, continuous electrocardiography at the DII derivation, heart rate, peripheral oxygen saturation (SpO_2_), esophageal temperature, pulse plethysmography, CO_2_ tension at the end of expiration (ETCO_2_), CO_2_ inspired tension, RR, systolic, diastolic, and mean arterial pressures, and minimum inspiratory and expiratory alveolar concentrations of the anesthetic agent were recorded continuously.

The system of anesthesia and mechanically controlled ventilation (Dameca Siesta Beasy) allowed PEEP, RR, peak airway pressure (Ppeak,_RS_), plateau airway pressure (Pplat,_RS_) by end‐inspiratory pauses (3 s), and *V*_T_ to be monitored. The respiratory system driving airway pressure (Δ*P*,_RS_) was calculated using the Pplat—PEEP. Mechanical power (MP) was calculated using the following formula (Serpa Neto et al., 2018):

$\mathrm{MP}=0.098\times \mathrm{RR}\times V_{T}\times \left[P\text{peak}{,}_{\mathrm{RS}}-\left(P\text{plat}{,}_{\mathrm{RS}}–\text{PEEP}\right)/2\right]$

The percentage fraction of dead space (*V*d/*V*t) was calculated according to available data using the adapted Enghoff equation (*V*d/*V*t) = (PaCO_2_ − ETCO_2_)/PaCO_2_ and the result was multiplied by 100 (Doorduin et al., 2016).

Arterial blood samples (1 ml) were collected in the same femoral artery catheter used for arterial pressure measurements and were immediately analyzed using an i‐STAT Wireless System (Abbot Laboratories) with CG4+ cartridges at the INITIAL and FINAL timepoints. The following parameters were assessed: hydrogen ion potential (pH), arterial partial pressure of oxygen (PaO_2_), arterial partial pressure of carbon dioxide (PaCO_2_), bicarbonate concentration (HCO_3_^−^), and lactate levels.

### 2.5 Echocardiography

All echocardiographic examinations were performed by one experienced examiner (A.L.S.T.) on both sides of the chest using an M‐Turbo system (FUJIFILM SonoSite) equipped with a 1.0–5.0 MHz phased array transducer (Px10, FUJIFILM SonoSite). The reference values for this species were based on Boon(Boon, 2011) for non‐sedated animals and Locatelli et al. (2011) for sedated animals.

Using M‐mode, through the right parasternal projection, at the cross‐section of the left ventricle and at the height of the papillary muscles, the following measurements were recorded: interventricular septum at the end of diastole (IVSd), diameter of the left ventricle at the end of diastole (LVIDd), thickness of the free wall of the left ventricle in diastole (LVPWd), aortic root (Ao), and left atrium (LA). Measurements calculated using this equipment included stroke volume (SV), fractional shortening (FS = 100 × [LVIDd−LVIDs]/LVIDd), and ejection fraction (EF). The end‐diastolic and end‐systolic left ventricular volumes (LVVd and LVVs, respectively) used in the following calculations were obtained using the Teichholz formula, as described by Hallowell et al. (2012) and Leroux et al. (2012). Left ventricular EF and SV were obtained using the following standard formulas: EF = [(LVVd−LVVs)/LVVd] × 100 and SV = LVVd−LVVs. Movements and functions of the aortic and mitral valves were inspected at the longitudinal right parasternal window. Using the same acoustic window, spectral Doppler of the pulmonary artery was performed through the cross section of the cardiac base, and the maximum pulmonary flow velocity (PVF Vel) was measured (Boon, 2011).

The speed of the mitral E wave and mitral A wave was obtained in the left parasternal window using spectral Doppler through the apical section of the four chambers. The ratio of mitral E and A (E/A mitral) waves was calculated using equipment software. The morphology of the mitral and tricuspid valves, as well as the flow direction through color Doppler, were also inspected. The aortic flow gradient (GrFAo) and heart rate were evaluated by obtaining images through the apical sections of the five chambers.

### 2.6 Statistical analysis

We did not perform a formal sample size calculation a priori; instead, we used all available sheep (10 animals). The primary outcome was Δ*P*,_RS_. Taking into account the Δ*P*,_RS_ difference in the G2 group at INITIAL and FINAL, the calculated effect size was 1.50. Thus, we performed a post hoc analysis to calculate the power achieved (1 − β err prob = 0.88), which was adequate.

Each variable was tested for normal distribution using the Shapiro–Wilk test. Data are presented as mean ± SD. The results were assessed using two‐way ANOVA, followed by a post hoc Holm–Sidák multiple comparisons test for differences among groups and sampling times. Correlation analyses were performed using Spearman (*r*) based on the change from INITIAL to FINAL, considering the respiratory mechanical variables. The significance for all tests was assumed to be *p* < 0.05. All statistical analyses were performed using GraphPad Prism version 9.2.0 (GraphPad Software).

## 3 RESULTS

### 3.1 Cardiovascular monitoring and echocardiography

Systolic, diastolic, and mean arterial pressures increased over time in all groups (*p* < 0.0001 for all) (Table 1); however, at INITIAL, diastolic and mean arterial pressures were higher in G4 than in G1 (*p* = 0.045, mean difference 11.11, 95% CI 22.11–0.10; and *p* = 0.035, mean difference 12.22, 95% CI 23.90–0.54, respectively).

**TABLE 1:** Hemodynamic and echocardiography parameters at initial and final times of procedures

Parameter	Group (mm Hg)	Initial	Final	*p* value		
				Time effect	Group effect	Interaction
SAP (mm Hg)				0.0001	0.134	0.211
	G1 (0)	67.6 ± 8.4	93.0 ± 19.8	^a^		
	G2 (10)	80.0 ± 14.9	95.7 ± 11.0	^a^		
	G3 (12)	71.4 ± 6.8	99.3 ± 3.7	^a^		
	G4 (15)	81.9 ± 12.2	96.0 ± 9.6	^a^		
DAP (mm Hg)				0.0001	0.050	0.163
	G1 (0)	44.7 ± 5.5	61.6 ± 16.1	^a^		
	G2 (10)	52.9 ± 7.9	63.9 ± 7.3	^a^		
	G3 (12)	46.9 ± 6.0	68.9 ± 3.0	^a^		
	G4 (15)	55.8 ± 10.3^b^	66.6 ± 5.7	^a^	vs G1	
MAP (mm Hg)				0.0001	0.081	0.108
	G1 (0)	54.2 ± 6.5	74.0 ± 16.4	^a^		
	G2 (10)	64.2 ± 9.9	76.7 ± 7.5	^a^		
	G3 (12)	56.8 ± 5.5	80.3 ± 3.0	^a^		
	G4 (15)	66.4 ± 10.7^b^	76.7 ± 6.5	^a^	vs G1	
HR (bpm)				0.0001	0.445	0.227
	G1 (0)	128 ± 17	99 ± 21	^a^		
	G2 (10)	141 ± 16	106 ± 17	^a^		
	G3 (12)	128 ± 32	120 ± 29			
	G4 (15)	128 ± 18	113 ± 24			
SV (ml)				0.0015	0.870	0.965
	G1 (0)	35.8 ± 12.1	28.2 ± 8.7			
	G2 (10)	35.5 ± 13.4	25.3 ± 7.6			
	G3 (12)	35.8 ± 6.9	28.7 ± 7.8			
	G4 (15)	38.2 ± 2.1	29.0 ± 12.3			
CO (l/min)				0.005	0.156	0.425
	G1 (0)	3.1 ± 0.7	2.7 ± 0.5			
	G2 (10)	3.8 ± 1.0	2.7 ± 0.6			
	G3 (12)	3.7 ± 1.0	3.5 ± 1.2			
	G4 (15)	3.9 ± 0.9	2,8 ± 1.0			
EF (%)				0.069	0.006	0.529
	G1 (0)	63.3 ± 8.1	62.1 ± 11.2			
	G2 (10)	71.4 ± 11.0	71.1 ± 12.1			
	G3 (12)	65.9 ± 6.3	59.7 ± 5.8^c^		vs G2	
	G4 (15)	65.6 ± 6.5	58.1 ± 6.9^c^		vs G2	
FS (%)				0.1227	0.0054	0.620
	G1 (0)	34.70 ± 5.19	33.60 ± 8.39			
	G2 (10)	41.07 ± 11.74	41.14 ± 12.26			
	G3 (12)	35.62 ± 4.93	31.20 ± 3.68^c^		vs G2	
	G4 (15)	35.61 ± 4.79	29.71 ± 5.11^c^		vs G2	
IVSd (cm)				0.502	0.040	0.335
	G1 (0)	0.817 ± 0.099	0.828 ± 0.129			
	G2 (10)	0.899 ± 0.144	0.961 ± 0.145			
	G3 (12)	0.870 ± 0.085	0.931 ± 0.087			
	G4 (15)	0.899 ± 0.136	0.838 ± 0.125			
LVIDd (cm)				0.163	0.085	0.186
	G1 (0)	3.447 ± 0.747	3.019 ± 0.809			
	G2 (10)	3.341 ± 0.639	3.160 ± 0.541			
	G3 (12)	3.464 ± 0.451	3.744 ± 0.569^b^		vs G1	
	G4 (15)	3.722 ± 0.147	3.341 ± 0.279			
LVPWd (cm)				0.069	0.636	P = 0.0029
	G1 (0)	1.020 ± 0.201	0.963 ± 0.224			
	G2 (10)	1.111 ± 0.231	0.798 ± 0.167	^a^		
	G3 (12)	1.127 ± 0.236	0.943 ± 0.236			
	G4 (15)	0.924 ± 0.132	1.123 ± 0.217^c^		vs G2	
LA/Ao				0.111	0.070	0.200
	G1 (0)	1.226 ± 0.166	1.277 ± 0.351			
	G2 (10)	1.172 ± 0.138	1.106 ± 0.141			
	G3 (12)	1.198 ± 0.193	1.416 ± 0.252^c^		vs G2	
	G4 (15)	1.138 ± 0.099	1.237 ± 0.114			
E/A Mitral				0.190	0.701	0.489
	G1 (0)	1.226 ± 0.210	1.007 ± 0.271			
	G2 (10)	1.184 ± 0.275	1.162 ± 0.203			
	G3 (12)	1.156 ± 0.270	1.147 ± 0.176			
	G4 (15)	1.108 ± 0.204	1.069 ± 0.220			
PVF Vel (cm/s)				0.0001	0.168	0.353
	G1 (0)	67.3 ± 18.7	46.8 ± 12.3	^a^		
	G2 (10)	64.7 ± 14.7	49.44 ± 14.7	^a^		
	G3 (12)	67.0 ± 19.9	41.5 ± 11.9	^a^		
	G4 (15)	69.1 ± 16.4	43.6 ± 16.4	^a^		
GrFAo (mm Hg)				0.0001	0.953	0.610
	G1 (0)	3.19 ± 1.06	1.97 ± 1.13	^a^		
	G2 (10)	3.34 ± 1.01	1.68 ± 0.83	^a^		
	G3 (12)	2.85 ± 1.36	2.14 ± 1.25			
	G4 (15)	3.30 ± 1.05	2.05 ± 1.14	^a^		

*Note*: Hemodynamic variables obtained at INITIAL and FINAL times of procedures, expressed as means ± standard deviation (SD) of 10 animals in each group. Data were assessed with 2‐way ANOVA and post hoc Holm–Sidák multiple comparisons test (*p* < 0.05).

Abbreviations: CO, cardiac output; DAP, diastolic arterial pressure; E/A Mitral, ratio between E and A mitral waves; EF, ejection fraction; FS, fractional shortening; GrFAo, aortic flow gradient; HR, heart rate; IVSd, diameter of the interventricular septum in diastole; LA/Ao, ratio between the diameter of the left atrium and the aorta; LVIDd, left ventricular internal diameter end diastole; LVPWd, left ventricular posterior wall in diastole; MAP, mean arterial pressure; PVF Vel, maximum pulmonary flow velocity; SAP, systolic arterial pressure; SV, stroke volume.

^a^ Difference between INITIAL and FINAL in the same group.

^b^ vs G1.

^c^ vs G2.

The HR, SV, CO, EF, FS, LVIDd, LVPWd, PVF Vel, and GrFAo decreased over time in all groups (Table 1). No major differences in HR, SV, CO, IVSd, E/A mitral ratio, and GrFAo were observed among the groups at INITIAL or FINAL. At FINAL, EF and FS were lower in G3 and G4 than in G2 (*p* = 0.039, mean difference − 11.38, 95% CI −0.07 to −22.68; *p* = 0.015, mean difference − 13.05. 95% CI −1.74 to −24.36; *p* = 0.039, mean difference − 9.94, 95% CI −0.07 to −19.80; *p* = 0.015, mean difference − 11.43, 95% CI −1.57 to −21.30, respectively). The LVIDd was higher in G3 than in G1 (*p* = 0.031, mean difference 0.72, 95% CI 1.40–0.04), and the LVPWd was higher in G4 than in G2 (*p* = 0.007, mean difference 0.32, 95% CI 0.58–0.06). The LA/Ao ratio was higher in G3 than in G2 (*p* = 0.009, mean difference 0.31, 95% CI 0.56–0.05).

### 3.2 Ventilatory monitoring and blood gas analysis

In addition to G1, Ppeak,_RS_, Pplat,_RS_, and Δ*P*,_RS_ increased over time in the other groups (Table 2). *V*_T_ did not differ over time or among the groups. At FINAL, Ppeak,_RS,_ and Δ*P*,_RS_ were higher in G3 and G4 than in G1 (*p* = 0.004, mean difference 5.0, 95% CI 8.8–1.1, *p* = 0.002, mean difference 5.4, 95% CI 9.28–1.51; *p* = 0.002, mean difference 4.60, 95% CI 7.91–1.28, and *p* < 0.001, mean difference 5.4, 95% CI 8.7–2.0, respectively). Pplat,_RS_ was higher in G3 than in G1 (*p* < 0.001, mean difference −4.8, 95% CI −8.0 to −1.5). In addition, Pplat,_RS_ was higher in G4 than in G1 and G2 (*p* < 0.001, mean difference − 5.5, 95% CI −8.7 to −2.2, and *p* = 0.049, mean difference − 3.1, 95% CI −6.3 to 0.1, respectively), whereas MP was only higher in G3 than G1 (*p* = 0.019, mean difference −1.8, 95% CI −3.5 to −0.2) but did not differ over time. At FINAL, *V*d/*V*t was higher in G3 and G4 than in G1 (*p* = 0.016, mean difference −9.5, 95% CI −17.9 to −1.2; *p* = 0.027, mean difference −8.7, 95% CI −17.1 to −0.4, respectively).

**TABLE 2:** Ventilatory parameters at initial and final times of procedures

Parameter	Groups (mm Hg)	INITIAL	FINAL	*p* value		
				Time effect	Group effect	Interaction
MAC insp				0.956	0.382	0.348
	G1 (0)	1.14 ± 0.15	1.17 ± 0.20			
	G2 (10)	1.12 ± 0.17	1.13 ± 0.30			
	G3 (12)	1.01 ± 0.14	1.11 ± 0.11			
	G4 (15)	1.13 ± 0.24	1.00 ± 0.22			
MAC exp				0.0002	0.698	0.880
	G1 (0)	0.89 ± 0.16	0.98 ± 0.15			
	G2 (10)	0.84 ± 0.18	1.00 ± 0.23			
	G3 (12)	0.80 ± 0.08	0.96 ± 0.12			
	G4 (15)	0.82 ± 0.16	0.97 ± 0.15			
*V*_T_ (ml/kg)				0.735	0.087	0.960
	G1 (0)	15.3 ± 0.5	15.5 ± 1.0			
	G2 (10)	15.2 ± 0.4	15.5 ± 1.5			
	G3 (12)	15.3 ± 0.6	15.3 ± 1.2			
	G4 (15)	14.6 ± 1.6	14.5 ± 2.0			
Ppeak,_RS_ (cmH_2_O)				0.0001	0.012	0.094
	G1 (0)	14.3 ± 2.6	14.9 ± 3.3			
	G2 (10)	14.5 ± 2.8	18.2 ± 4.0	^a^		
	G3 (12)	15.3 ± 3.1	19.9 ± 2.6^b^	^a^	vs G1	
	G4 (15)	14.8 ± 3.6	20.3 ± 3.3^b^	^a^	vs G1	
Pplat,_RS_ (cmH_2_O)				0.0001	0.0039	0.013
	G1 (0)	13.6 ± 2.6	14.1 ± 2.5			
	G2 (10)	13.2 ± 1.6	16.5 ± 2.3	^a^		
	G3 (12)	13.7 ± 2.6	18.9 ± 2.9^b^	^a^	vs G1	
	G4 (15)	13.9 ± 2.9	19.6 ± 3.4^c^	^a^	vs G1 and G2	
Δ*P*,_RS_ (cmH_2_O)				0.0001	0.006	0.018
	G1 (0)	10.7 ± 2.9	11.2 ± 2.5			
	G2 (10)	10.5 ± 1.8	13.6 ± 2.3	^a^		
	G3 (12)	10.8 ± 2.6	15.8 ± 2.9‑	^a^	vs G1	
	G4 (15)	11.0 ± 3.0	16.6 ± 3.4^b^	^a^	vs G1	
MP (J/min)				0.002	0.069	0.303
	G1 (0)	5.05 ± 0.89	5.03 ± 1.70			
	G2 (10)	5.90 ± 3.94	6.88 ± 2.97	^a^		
	G3 (12)	5.49 ± 1.09	6.90 ± 1.20^b^	^a^	vs G1	
	G4 (15)	5.10 ± 1.29	6.28 ± 1.26	^a^		
*V*%				0.023	0.206	0.383
	G1 (0)	1.84 ± 0.39	1.72 ± 0.38			
	G2 (10)	1.80 ± 0.48	1.60 ± 0.28			
	G3 (12)	1.52 ± 0.21	1.56 ± 0.26			
	G4 (15)	1.86 ± 0.5	1.51 ± 0.20	^a^		
*V*d/*V*t (%)				0.631	0.673	0.004
	G1 (0)	47.6 ± 7.95	37.7 ± 9.27	^a^		
	G2 (10)	45.1 ± 3.74	44.0 ± 5.90			
	G3 (12)	41.7 ± 5.01	47.3 ± 5.47^b^		vs G1	
	G4 (15)	43.9 ± 4.74	46.5 ± 4.61^b^		vs G1	

*Note*: Ventilation variables obtained at INITIAL and FINAL times of procedures, expressed as means ± standard deviation (SD) of 10 animals in each group. Data were assessed with two‐way ANOVA and post hoc Holm–Sidák multiple comparisons test (*p* < 0.05).

Abbreviations: MAC exp, minimum alveolar concentration in expiration; MAC insp, minimum alveolar concentration in inspiration; MP, mechanical power; Ppeak,_RS_, peak pressure; Pplat,_RS_, plateau pressure; *V*%, vaporized volume of anesthetic gas; *V*d/*V*t, percentage fraction of dead space; *V*_T_, tidal volume; Δ*P*,_RS_, distension pressure.

^a^ Difference between INITIAL and FINAL in the same group.

^b^ vs G1.

^c^ vs G1 and G2.

PaCO_2_ and HCO_3_ levels increased over time in all groups (Table 3). At FINAL, PaCO_2_ was higher in G3 and G4 than in G1 (*p* < 0.001 for both; mean difference 13.26, 95% CI 21.8–4.7, and mean difference 13.49, 95% CI 22.0–4.9, respectively). PaO_2_/FiO_2_ decreased over time in all groups except G1, which showed an increase, and PaO_2_/FiO_2_ was lower in G4 than in G1 (*p* = 0.008, mean difference −95.5. 95% CI −18.12 to −172.9). Lactate and body temperature decreased over time in all groups (*p* < 0.0001).

**TABLE 3:** Blood gas analysis at initial and final times of procedures

	Groups (mm Hg)	Initial	Final	*p* value		
				Time effect	Group effect	Interaction
pHa				0.848	0.683	0.048
	G1 (0)	7.30 ± 0.05	7.35 ± 0.07			
	G2 (10)	7.31 ± 0.03	7.31 ± 0.05			
	G3 (12)	7.32 ± 0.06	7.28 ± 0.05			
	G4 (15)	7.32 ± 0.03	7.29 ± 0.04			
PaCO_2_ (mm Hg)				0.0001	0.019	0.003
	G1 (0)	56.1 ± 5.2	57.1 ± 8.3			
	G2 (10)	55.5 ± 5.8	63.1 ± 6.9	^a^		
	G3 (12)	54.8 ± 6.4	70.3 ± 7.1^b^	^a^	vs G1	
	G4 (15)	54.9 ± 3.6	70.6 ± 10.6^b^	^a^	vs G1	
PaO_2_/FiO_2_				0.0004	0.212	0.021
	G1 (0)	379 ± 55	387 ± 65			
	G2 (10)	390 ± 65	363 ± 52			
	G3 (12)	404 ± 73	315 ± 70	^a^		
	G4 (15)	396 ± 65	292 ± 60^b^	^a^	vs G1	
HCO_3_ (mmol/L)				0.0001	0.062	0.755
	G1 (0)	27.59 ± 2.19	31.80 ± 2.15	^a^		
	G2 (10)	27.98 ± 1.76	32.18 ± 1.44	^a^		
	G3 (12)	28.52 ± 2.67	33.60 ± 2.35	^a^		
	G4 (15)	28.63 ± 2.21	34.04 ± 2.44	^a^		
Lactate (mmol/L)				0.0001	0.778	0.780
	G1 (0)	2.11 ± 1.04	0.73 ± 0.48	^a^		
	G2 (10)	2.56 ± 1.08	0.65 ± 0.51	^a^		
	G3 (12)	2.16 ± 1.09	0.55 ± 0.27	^a^		
	G4 (15)	2.26 ± 0.99	0.60 ± 0.25	^a^		
Body temperature (°C)				0.0001	0.148	0.987
	G1 (0)	37.4 ± 0.6	35.9 ± 0.5	^a^		
	G2 (10)	37.1 ± 0.7	35.4 ± 0.7	^a^		
	G3 (12)	37.2 ± 0.5	35.6 ± 0.7	^a^		
	G4 (15)	37.5 ± 0.9	35.9 ± 0.7	^a^		

*Note*: Blood gas analysis variables obtained at INITIAL and FINAL times of procedures, expressed as means ± standard deviation (SD) of 10 animals in each group. Data were assessed with two‐way ANOVA and post hoc Holm–Sidák multiple comparisons test (*p* < 0.05).

Abbreviations: HCO_3_^−^, hydrogen carbonate in blood; PaCO_2_, partial pressure of arterial carbon dioxide; PaCO_2_/FiO_2_, ratio between partial pressure of oxygen in arterial blood and fraction of inspired oxygen; pHa, arterial potential of hydrogen.

^a^ vs G1.

^b^ Difference between INITIAL and FINAL in the same group.

### 3.3 Correlations

**FIGURE 2:**
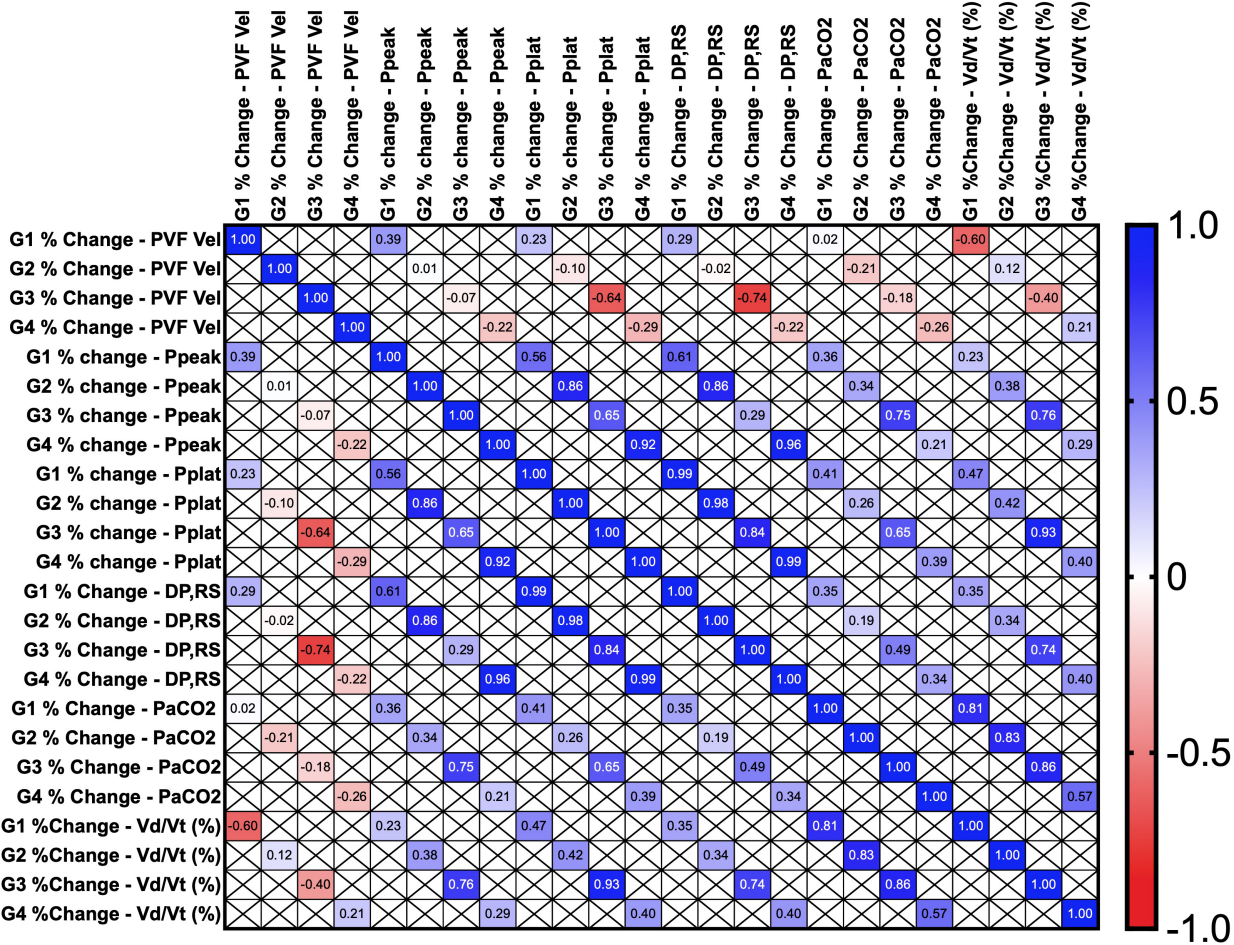
Heatmap of Spearman's correlation (*r*) between percentage change in ventilatory, gasometric, and echocardiographic variables between INITIAL and FINAL timepoints for each group. The *r* values are contained within each square and follow the colourimetric scale on the right.

Spearman (*r*) correlation analysis of the percentage change in each group (Table 4, Figure 2) showed that PVF Vel decreased, and Δ*P*,_RS_ increased in G3 (*r* = −0.740; *p* = 0.018). *V*d/*V*t positively correlated with Ppeak,_RS_ (*r* = 0.762, *p* = 0.037), Pplat,_RS_ (*r* = 0.928, *p* = 0.002), and Δ*P*,_RS_ (*r* = 0.738, *p* = 0.046). Vd/Vt was positively correlated with PaCO_2_ in G1, G2, and G3 (*r* = 0.809, *p* = 0.022; *r* = 0.833, *p* = 0.015; *r* = 0.857, *p* = 0.010, respectively).

**TABLE 4:** Spearman correlation (*r*) between percentage change in some ventilatory, gasometric, and echocardiographic variables and their respective *p* values between INITIAL and FINAL times for each group

	Ppeak,_RS_		Pplat,_RS_		Δ*P*,_RS_		PaCO_2_	
	*r*	*p*	*r*	*p*	*r*	*p*	*r*	*p*
G1 PVF Vel	0.394	0.264	0.234	0.514	0.293	0.409	0.018	0.973
G2 PVF Vel	0.006	0.992	−0.097	0.791	−0.024	0.953	−0.212	0.560
G3 PVF Vel	−0.067	0.857	−0.638	0.052	−0.740	0.018	−0.176	0.624
G4 PVF Vel	−0.224	0.537	−0.289	0.414	−0.220	0.539	−0.261	0.470
G1 Vd/Vt	0.228	0.590	0.466	0.243	0.349	0.395	0.809	0.022
G2 Vd/Vt	0.383	0.347	0.419	0.301	0.337	0.410	0.833	0.015
G3 *V*d/Vt	0.762	0.037	0.928	0.002	0.738	0.046	0.857	0.010
G4 *V*d/*V*t	0.286	0.501	0.395	0.334	0.405	0.327	0.571	0.151

*Note*: Data were assessed with Spearman (*r*) correlation (*p* < 0.05) between percentage change in each variable for INITIAL and FINAL time of each respective group.

Abbreviations: Ppeak,RS, peak pressure; Pplat,RS, plateau pressure; ΔP,RS, distension pressure; PaCO_2_, partial pressure of arterial carbon dioxide; Vd/Vt, percentage fraction of dead space; PVF Vel, maximum pulmonary flow velocity.

**FIGURE 3:**
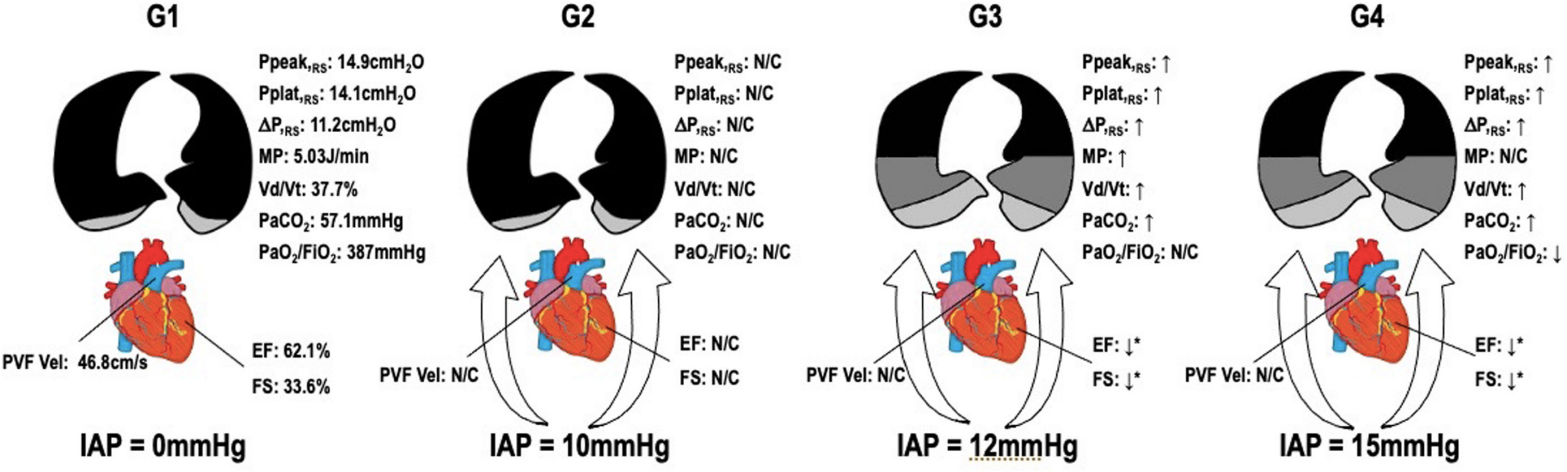
Summary of the results according to the group and the respective intraabdominal pressure (IAP) at FINAL. In G1, mean values are presented after 1 h at IAP = 0 mm Hg. In G2 to G4, variables are relative to values obtained in G1: N/C, no changes; ↑, increased; ↓, decreased. ↑* is relative to G2. Ppeak,_RS_, peak airway pressure; Pplat,_RS_, plateau airway pressure; DP,_RS_, driving airway pressure; MP, mechanical power; *V*d/*V*t, dead space ventilation; PaCO_2_, partial pressure of carbon dioxide; PaO_2_/FiO_2_, partial pressure of oxygen divided by the inspired fraction of oxygen; EF, ejection fraction; FS, fractional shortening; PVF Vel, pulmonary venous flow velocity.

A summary of the relevant findings and interactions is presented in Figure 3.

## 4 DISCUSSION

In this study, we found that (iData Research Inc, 2020) Ppeak,_RS_, Pplat,_RS_, Δ*P*,_RS_, MP, and *V*d/*V*t were higher in groups with IAP settled at 12 and 15 mm Hg for the same tidal volume (Malbrain et al., 2005) left ventricular function was also affected, as observed by decreased EF and FS, in groups with IAP settled at 12 and 15 mm Hg (Atkinson et al., 2017) although PVF Vel correlated negatively with Pplat,_RS_ and Δ*P*,_RS_ in G3, it did not change across the different groups; (Koivusalo & Lindgren, 2000) although PaCO_2_ levels were higher and PaO_2_/FiO_2_ levels were lower in the group with highest IAP (15 mm Hg), these changes were not followed by changes in arterial pH. Cardiorespiratory changes were observed in IAP levels adjusted to 12 and 15 mm Hg, which are commonly observed in laparoscopic procedures in humans (Percie du Sert et al., 2020; Massone, 2011). The present study demonstrates the importance of monitoring respiratory mechanics in precluding changes in left cardiac function. This experimental study may have an impact on cardiac performance and respiratory mechanics in patients with poor baseline condition before abdominal surgery, as seen in prognostic and complications of individuals chronic obstructive pulmonary disease (COPD) submitted to laparoscopic procedures (Liao et al., 2019), as well as patients with cardiac and pulmonary comorbidities and those who undergo total gastrectomy and combined resection should be considered at high risk for pneumonia, pleural effusion, or pulmonary embolism (Ntutumu et al., 2016).

The sheep model is widely used because the mechanisms related to the formation of atelectasis, pulmonary shunt, and reduced oxygenation, as well as hypoxic pulmonary vasoconstriction, are like those in humans (Underwood et al., 2015). The IAP levels used here are closer to those used in humans, not exceeding 15 mm Hg (Sharma et al., 1996).

The echocardiographic parameters of systolic and diastolic left ventricular performance in young adult sheep can be reliably extrapolated to adult humans because the dimensional and functional parameters are within human reference values (Boon, 2011; Poser et al., 2013). The echocardiographic examinations were performed following the techniques described and recommended by the American College of Veterinary Internal Medicine (Thomas & Weyman, 1991), American Society of Echocardiography (Locatelli et al., 2011), and Abduch et al. (2014).

### 4.1 Cardiovascular monitoring and echocardiography

In humans, during laparoscopy, according to Wahba et al. (1995) and Oliveira (2005) even small IAP values (10 mm Hg) can lead to hemodynamic alterations such as a decrease in CO. However, CO did not significantly reduce over time (Table 1), which has also been observed in sedated and anesthetized sheep (Izwan et al., 2018). Accordingly, in a healthy pig model, IAP levels of 12, 18, and 22 mm Hg at a PEEP of 5 cm H_2_O did not decrease CO. The decrease in CO was mainly observed after increasing the PEEP levels (Regli et al., 2012), which did not occur in this study.

Even though MAP, SAP, and DAP (Table 1) demonstrated a hypotensive status at INITIAL in some groups, we hypothesized it is due to water and food restriction required for anesthetic and surgical procedures in sheep, and not as much for the anesthetic protocol utilized since this was maintained for the entire 60‐min period and blood pressure values increased. Also, the interaction between fentanyl and isoflurane has shown not to clinically affect the cardiorespiratory stability or postoperative recovery in anesthetized sheep (Funes et al., 2015) Also, regarding those findings, the only intervention during procedures was fluid therapy with crystalloid fluids to keep vein open, which was enough to increase these parameters significantly in all groups at FINAL. It has been shown that pneumoperitoneum increases MAP and systemic vascular resistance and may decrease CO due to abdominal aortic compression, along with neuroendocrine effects (Atkinson et al., 2017; O'Leary et al., 1996; O'Malley & Cunningham, 2001). Additionally, the increase in IAP exerts a mechanical compression effect on the venous, arterial, and capillary abdominal vasculature (O'Malley & Cunningham, 2001) which can generate an increase in MAP similarly to what would occur in peripheral vasoconstriction.

Considering the decrease in HR (Table 1) at FINAL, during anesthesia maintenance no nociceptive stimulus was performed. Also, fentanyl was given in a bolus prior to continuous IV administration, being this a contributing factor also found by Funes et al. (2015) even though the use of balanced general anesthesia a common practice for laparoscopy (Lehavi et al., 2018). According to Cohen et al. (2003), this decrease also occurs by the activation of vagal tone with peritoneal distention.

Other echocardiographic changes were observed, such as decreased FS and EF (Table 1) in groups with higher IAP (G3 and G4), secondary to poor preload, increased afterload, or decreased contractility (Aurigemma et al., 2002; Boon, 2011; Kittleson, 1994a, 1994b; Kittleson & Kienle, 1998; O'Malley & Cunningham, 2001; Silva et al., 2008). According to Kittleson and Kienle (1998), Silva et al. (2008) and Lago et al. (2009) these changes are surrogates for poor ventricular function. Values of LVPWd and LVIDd (Table 1), which are used to calculate EF and FS, were close to the values reported by Boon (2011), Hallowell et al. (2012), and Aleixo (2019) and higher than those found by Locatelli et al. (2011) explaining the mentioned changes.

Pulmonary artery flow velocity is determined by pulsed‐wave Doppler (PWD), with its peak velocity and peak pressure occurring in mid systole. In the normal pulmonary circulation wave reflection is not evident but can be seen in the presence of elevated pulmonary vascular impedance and decreased compliance. The reflected pressure wave is additive to forward pressure and imposes an added load on right ventricle ejection while the reflected flow wave causes a corresponding reduction in flow velocity, as seen in our study. Also, several patterns of abnormal pulmonary artery PWD profiles have been described and they represent different magnitudes and timing of the reflected wave and may also be related to stroke volume and heart rate (Hu et al., 2012).

PVF Vel and GrFAo (Table 1) were within normal values, but decreased in all groups at FINAL, which, according to Mohamad Ali et al. (2019) is due to flow velocities decrease along with SV. In this study, the animals used had no previous cardiorespiratory alterations, leading to these findings reflecting no impairment of the right ventricle function, which was the opposite observed by Chemonges et al. (2020) in sheep with smoke‐induced lung injury during extracorporeal membrane oxygenation presenting decreasing values of right ventricular stroke work index among other findings over time. Therefore, we found that injured lungs generate an impact between right and left ventricular function, since in our study with healthy individuals, gradual respiratory mechanics impairment (Table 2) was reflected in decreased left heart function.

### 4.2 Ventilatory monitoring and blood gas analysis

Respiratory mechanics worsened in animals with increased IAP at the FINAL (Table 2). According to Valenza et al. (2010), IAP can cause stiffening of the chest wall and components, abdominal wall, and diaphragm muscle, which in turn increases the transmural pressure and reflects the increase in airway pressure (Ppeak,_RS_ and Pplat,_RS_) during volume‐controlled ventilation.

Δ*P*,_RS_ levels increased at FINAL in all groups. According to Pelosi et al. (2021) in humans, Δ*P*,_RS_ is considered to be within a safe range when it is <13 cm H_2_O, and the mean values for G3 and G4 were above that. Accordingly, Serpa Neto et al. (2018) state that a high Δ*P*,_RS_ reflects decreased respiratory system compliance or excessive *V*_T_, which can induce lung injury due to excessive stretching. In this study, *V*_T_ and PEEP were not altered; therefore, the increase in Δ*P*,_RS_ can be explained by a higher IAP. This was also observed in the Vd/Vt values for G3 and G4 compared to G1. In this study, MP also increased in all groups except G1 at FINAL, which according to Pelosi et al. (2021) is a well‐recognized determinant of ventilator‐induced lung injury.

Regarding blood gas analysis, the greater the pneumoperitoneal pressure, the greater the tendency toward hypercarbia (Table 3). Values of HCO_3_^−^ showed a significant increase in all groups at FINAL, which corroborates the findings of Hikasa et al. (1998, 2000) and de Mattos Junior (2012) demonstrating that it can be considered a compensatory measure for the increase in PaCO_2_ observed previously. According to Chemonges (2020) the measurement of HCO_3_^−^ in the blood indicates the body's ability to handle additional amounts of organic acids.

The PaO_2_/FiO_2_ values were inversely proportional to the IAP. This increase causes diversion of blood in the pulmonary circulation to lower pressure regimes and, consequently, leads to a change in the relationship between ventilation and perfusion, increasing the shunt effect and dead‐space ventilation. These changes have been observed elsewhere (Abduch et al., 2014; Wahba et al., 1995). In this study, even with decreased values of PaO_2_/FiO_2_ in G3 and G4 at FINAL, lactate levels decreased in all groups, indicating adequate peripheral perfusion, which agrees with Hughes (2000).

### 4.3 Correlations

Finally, PVF Vel showed a negative correlation with Δ*P*,_RS_ in G3 (Table 4, Figure 2), which may reflect an association between flow velocities decreasing along with SV (Mohamad Ali et al., 2019) and decreased respiratory system compliance and/or lung volume expressed by higher values of Δ*P*,_RS_. Accordingly, in G3, a positive correlation was observed between *V*d/*V*t and Ppeak,_RS_, Pplat,_RS_, Δ*P*,_RS_, and PaCO_2_ (Table 4, Figure 2). In addition, *V*d/*V*t was positively correlated with PaCO_2_ in all groups, indicating that the dead space effect was sustained in groups with higher IAP, impairing ventilatory performance.

### 4.4 Limitations

We used nonpregnant ewes due to the facility availability. However, we may infer that some differences would occur. These differences would be related to animal's size and sex, drug consumption, and anesthesia composition.

The cranial location of the heart at FINAL with the animals in dorsal recumbency associated with the increase in IAP displacing the rumen was the main issue in assessing echocardiographic parameters through transthoracic echocardiography, as mentioned by other authors (Olsson et al., 2001). At INITIAL, echocardiography was done while the animals were awake, and thus caution should be raised when comparing INITIAL vs FINAL time points. However, the comparison of data at FINAL may not be influenced since all animals were submitted to similar regimen of anesthesia and neuromuscular blockade, and thus, this may not preclude our interpretation about cardiovascular performance across different IAP levels.

Respiratory acidosis at INITIAL was inherently to the ventilator protocol, which consisted of the application of tidal volume (15 ml/kg), positive end‐expiratory pressure (PEEP) of 3 cmH_2_O, and respiratory rate (RR) ranging between 6 and 10 bpm. This protocol did not allow changes in RR to keep PaCO_2_ in a certain level reflecting a limitation. Nevertheless, we can compare all animals at INITIAL and FINAL since they were under similar ventilatory protocol.

## 5 CONCLUSION

Impaired respiratory system mechanics and reduced left cardiac function were observed at IAP of 12 and 15 mm Hg, while no changes in maximum pulmonary artery flow velocity were detected. At a high IAP, respiratory mechanics should be monitored as an interplay to reduce left cardiac function.

## FUNDING INFORMATION

Support was provided solely from institutional and/or departmental sources.

## CONFLICT OF INTEREST

None.

## Supporting information


Table S1
