# Supplementary material for: Cardiorespiratory effects of different intraabdominal pressures in sheep: An experimental study
Source: Physiol Rep. 2022 Nov 10;10(21):e15506. doi: 10.14814/phy2.15506 (PMC9647340; doi:10.14814/phy2.15506)
Supplement: Supplementary file 1 — Table S1 [file PHY2-10-e15506-s001.docx]

Table S1 Relationships between each sheep and the corresponding group in the sequence in which they were submitted to the procedure with 15-day intervals between each round: G1 (0 mmHg), G2 (10 mmHg), G3 (12 mmHg), G4 (15 mmHg)

| **Animal identification** | **Group sequence** | | | |
| --- | --- | --- | --- | --- |
|  | **1st round** | **2nd round** | **3rd round** | **4th round** |
| 1 | G2 | G1 | G4 | G3 |
| 2 | G4 | G2 | G3 | G1 |
| 3 | G1 | G4 | G3 | G2 |
| 4 | G1 | G4 | G2 | G3 |
| 5 | G2 | G3 | G4 | G1 |
| 6 | G1 | G3 | G2 | G4 |
| 7 | G3 | G2 | G4 | G1 |
| 8 | G2 | G4 | G1 | G3 |
| 9 | G1 | G3 | G4 | G2 |
| 10 | G2 | G1 | G3 | G4 |
